# Ultrasound Assisted Synthesis of 4-(Benzyloxy)-*N*-(3-chloro-2-(substitutedphenyl)-4-oxoazetidin-1-yl) Benzamide as Challenging Anti-Tubercular Scaffold

**DOI:** 10.3390/molecules23081945

**Published:** 2018-08-03

**Authors:** Urja D. Nimbalkar, Julio A. Seijas, Rachna Borkute, Manoj G. Damale, Jaiprakash N. Sangshetti, Dhiman Sarkar, Anna Pratima G. Nikalje

**Affiliations:** 1 Maulana Azad P. G. and Research Centre, Dr. Rafiq Zakaria Campus, Rauza Baug, Aurangabad 431001, India; urjasatish@gmail.com; 2 Departamento de Química Orgánica, Facultad de Ciencias, Universidad of Santiago de Compostela, Alfonso X el Sabio, 27002 Lugo, Spain; julioa.seijas@usc.es; 3 Combichem-Bio Resource Centre, Division of Organic Chemistry, CSIR-National Chemical Laboratory, Pune 411008, Maharashtra, India; rachnabrkt@gmail.com (R.B.); d.sarkar@ncl.res.in (D.S.); 4 Department of Pharmaceutical Chemistry, Srinath College of Pharmacy, Aurangabad 431116, India; pharmalink1985@gmail.com; 5 Department of Pharmaceutical Chemistry, Dr. Rafiq Zakaria Campus, Y. B. Chavan College of Pharmacy, Aurangabad 431001, India; jnsangshetti@rediffmail.com

**Keywords:** green chemistry, ultra-sonication, azetidinone, anti-tubercular screening, cytotoxicity study, molecular docking, ADMET study

## Abstract

A series of ten novel derivatives of 4-(benzyloxy)-*N*-(3-chloro-2-(substituted phenyl)-4-oxoazetidin-1-yl) benzamide **6a**–**j** were synthesized in good yield from the key compound 4-(benzyloxy)-*N*′-(substituted benzylidene) benzo hydrazide, called Schiff ’s bases **5a**–**j**, by Staudinger reaction ([2 + 2] ketene-imine cycloaddition reaction) with chloro acetyl chloride in the presence of catalyst tri ethylamine and solvent dimethyl formamide (DMF), by using ultra-sonication as one of the green chemistry tools. All the synthesised compounds were evaluated for *in vitro* anti-tubercular activity against *Mycobacterium tuberculosis* (MTB) and most of them showed promising activity with an IC_50_ value of less than 1 µg/mL. To establish the safety, all the synthesized compounds were further tested for cytotoxicity against the human cancer cell line HeLa and all **6a**–**j** compounds were found to be non-cytotoxic in nature. The molecular docking study was carried out with essential enzyme InhA (FabI/ENR) of *Mycobacterium* responsible for cell wall synthesis which suggests that **6a** and **6e** are the most active derivatives of the series. The theoretical evaluation of cell permeability based on Lipinski’s rule of five has helped to rationalize the biological results and hence the synthesized azetidinone derivatives **6a**–**j** were also analyzed for physicochemical evaluation that is, absorption, distribution, metabolism, excretion, and toxicity (*ADMET*) properties and the results showed that all the derivatives could comply with essential features required for a potential lead in the anti-tubercular drug discovery process.

## 1. Introduction

Tuberculosis (TB) is one of the life threating disease caused by *Mycobacterium tuberculosis* (*MTB*), which has shown advanced mechanisms to evade host defense. Decades after the discovery of *MTB*, TB remains a major cause of morbidity and mortality in many developing countries. The latent infection of (MTB) has infected nearly one-third of the population all over the globe. Based on the report published by World Health Organization (WHO), globally 1.5 million people died and approximately 9 million people were diagnosed with TB in 2015 [1,2,3,4], and the number of new infected cases has continually been on rise up to 2018. The long duration of therapy and side effects of existing anti-tubercular drugs [5,6] leads to failure to cure TB and results in highly lethal, extremely expensive, and complicated to treat [7,8,9] conditions such as multi-drug resistant (MDR-TB) and extensively drug-resistant tuberculosis (XDR-TB). Recently, a more dangerous and incurable form of TB known as totally drug resistant tuberculosis (TDR) has also been reported [10,11,12,13]. Although several new compounds are currently in different stages of clinical trials [14,15,16,17,18], only a few drugs, such as bedaquiline, have been recently approved by the FDA for use in drug resistant TB [19,20]. Hence, it is crucial to develop new drugs which will not only effectively treat MDR and XDR tuberculosis conditions but also reduce the complexity and duration of the current therapeutic treatment.

The four-membered cyclic amides commonly known as azetidin-2-one or β-lactam occupy an eminent place in organic and medicinal chemistry since the structure of the renowned drug penicillin showed the presence of a β-lactam ring in it. The various potent biological activities of the drug penicillin are due to the presence of the β-lactam ring. This discovery lead to designing chemical moieties containing a β-lactam ring, and development of several novel methodologies for construction of several β-lactam rings comprising antibiotics such as carbapenams, cephalosporins, monobactams, and trinems [21,22,23]. Azetidinones are known to exhibit anti-tubercular [24,25,26,27,28,29], antibacterial, and antifungal activity [30,31].

**Figure 1:**
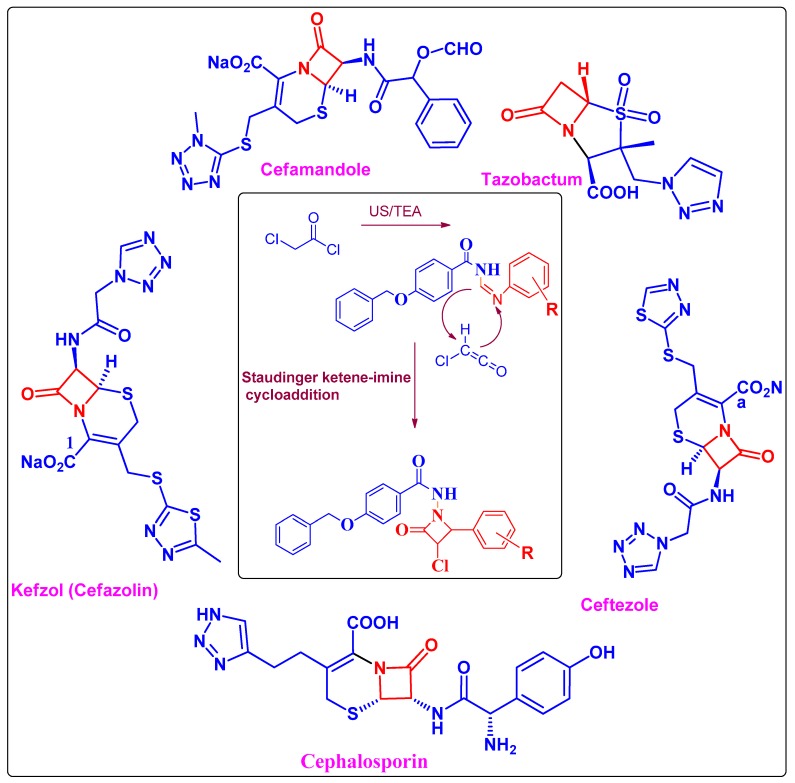
The design protocol for the synthesis of the target molecules.

The research protocol was designed by using the Staudinger ketene-imine cycloaddition reaction for the synthesis of the β-lactam ring, taking into consideration existing commercially available antibiotic drugs such as cefamandole, tazobactum, kefzol, ceftezole, and cephalosporin containing a β-lactam ring and NH-C=O group as an important pharmacophore. The biological activity of the β-lactam skeleton is generally believed to be associated with the chemical reactivity of its β-lactam ring; the oxo group is at 2nd position and on the substituent, especially at the nitrogen of the 2-azetidinone ring. In this research work ten derivatives of 4-(benzyloxy)-*N*′-(substituted benzylidene) benzo hydrazide, called Schiff’s bases **5a**–**j**, underwent a Staudinger ketene-imine cycloaddition reaction so as to get the final azetidin-2-one derivative namely 4-(benzyloxy)-*N*-(3-chloro-2-(substituted phenyl)-4-oxoazetidin-1-yl) benzamide **6a**–**j**. Hydrazide fused azetidin-2-one coupled with various substituted phenyl and heteryl rings may enhance anti-mycobacterial activity. The cyclization of synthesized Schiff’s base derivatives into a β-lactam containing azetidin-2-one derivatives may shows enhanced *in vitro* anti-tubercular activity. The designing protocol for the synthesis of target compounds is presented in Figure 1.

The shortcomings associated with existing methods of organic synthesis reported for the azetidin-2-one derivatives by a conventional method like refluxing at high temperature and stirring at room temperature are that it requires several hours for completion of the reaction, with a lesser amount of product yield, and it consumes more solvents, time, and electricity [32,33,34,35]. Green chemistry is a new branch of chemistry which has become a major motivation for organic chemists and druggists to develop an environmentally gentle path for synthesis of organic compounds of biological importance in which ultrasound assisted synrhesis of azetidin-2-one derivatives is now today’s method of choice for many researchers [36].

Ultrasonic-assisted organic synthesis (UAOS) has emerged as an eco-friendly technology in green chemistry [37,38]. The effects of ultrasound on organic reactions are attributed to cavitation, a physical process that creates, enlarges, and implodes gaseous and vaporous cavities in an irradiated liquid [39]. The cavitation induces very high local temperatures and pressures inside the bubbles (cavities), leading to a turbulent flow in the liquid and enhanced mass transfer. In the last decade, ultrasound irradiation is increasingly used as an alternative energy source to promote several organic transformations [40,41,42] in higher yields, shorter reaction times, and milder conditions, being considered a clean and useful protocol compared with traditional methods [43,44,45,46].

All the synthesized derivatives **6a**–**j** were screened for their *in vitro* anti-tubercular activity by XTT Reduction Menadione assay (XRMA) and cytotoxicity study by MTT assay.

To simulate the interaction between a protein and a ligand at the atomic level and to predict and demonstrate the performance of ligands in the binding site of target proteins [47], molecular docking study is a popular computational tool used in drug discovery. A major cell wall component of *M. tuberculosis* is mycolic acid and is hence among the various targets being explored for anti-tubercular activity; enzymes that are responsible for the inhibition of fatty acid synthesis are an attractive target for the new anti-tubercular agents. Enzymes-FAS-I and FAS-II in *Mycobacterium* catalyzed fatty acid synthesis; the enzyme enoyl-ACP (CoA) reductase (FabI/ENR/InhA) is an important enzyme in the FAS-II system [48] which is selected as a target enzyme for the study. The primary target for the preferred anti-tubercular agent isoniazid was inhA structural gene, (InhA) in *M. tuberculosis* which was identified as an NADH-dependent enoyl-ACP (CoA) reductase specific for chain elongation and a precursor of mycolic acids [49]. Hence, the synthesized derivatives **6a**–**j** were docked in the active site with essential enzymes InhA (FabI/ENR) of *Mycobacterium* responsible for cell wall synthesis.

Computational prediction of physicochemical parameters plays a crucial role for the improvement of pharmacokinetic properties of the most promising drug/lead candidates. To evaluate the drug-likeness and oral rate of bio-availability of the synthesized derivatives, the physicochemical parameters based on the Lipinski RO5 (Rule of Five) [50] were predicated by FAFdrug2.

## 2. Results and Discussion

### 2.1. Chemistry

**Scheme 1:**
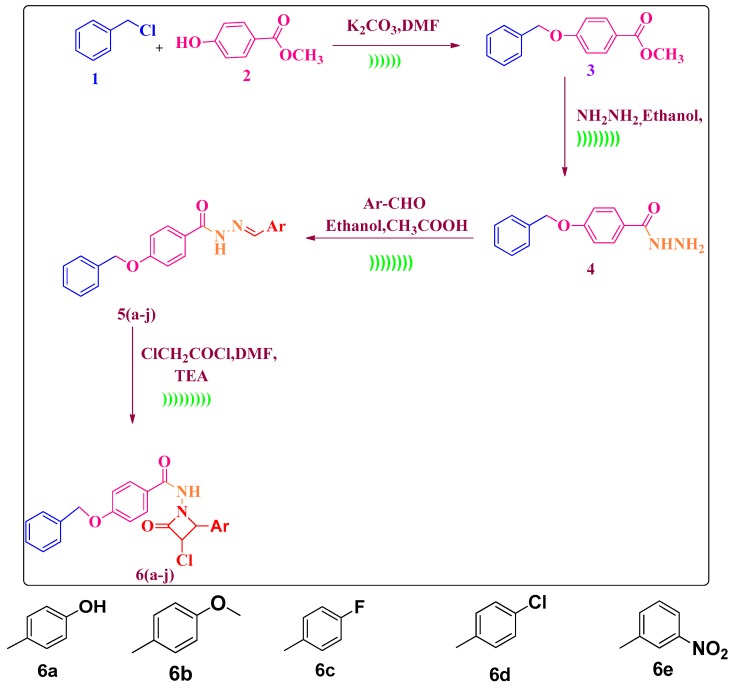

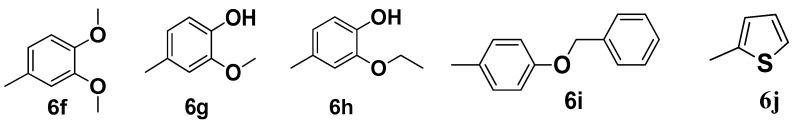
Synthesis of 4-(benzyloxy)-*N*-(3-chloro-2-(substituted phenyl)-4-oxoazetidin-1-yl) benzamide **6a**–**j**.

Herein, we are reporting the one pot synthesis of *4*-(benzyloxy)-*N*-(3-chloro-2-(substituted phenyl)-4-oxoazetidin-1-yl) benzamide **6a**–**j** as illustrated in Scheme 1.

The starting material methyl 4-(benzyloxy) benzoate **3** was synthesized by the reaction of benzyl chloride **1** and methyl 4-hydroxybenzoate **2** in potassium carbonate as a catalyst in solvent *N*,*N* dimethyl formamide, in an ultra-sonicator up to 4 h [51]. The compound **3** obtained in good yield in step I was treated further with hydrazine hydrate to get 4-(benzyloxy) benzohydrazide **4**. Schiff bases **5a**–**j** were obtained by condensation 4-(benzyloxy)benzohydrazide with various aromatic aldehydes. Schiff’s bases **5a**–**j** undergo cyclocondensation with chloro acetyl chloride by ultra-sonication in DMF, in the presence of triethylamine as a catalyst to give the final products **6a**–**j**.

Synthesis of reported azetidin-2-one derivatives by a conventional method like stirring at room temperature required 20–28 h and by refluxing at high temperature required 8–10 h for completion of the reaction; whereas by using a green chemistry tool like ultra-sonication the time of synthesis was reduced up to 2 h. The obtained products **6a**–**j** were recrystallized from ethanol and were obtained in excellent yield.

The physical characterization is as shown in Table S1 (provided in Supplementary File).

**Figure 2:**
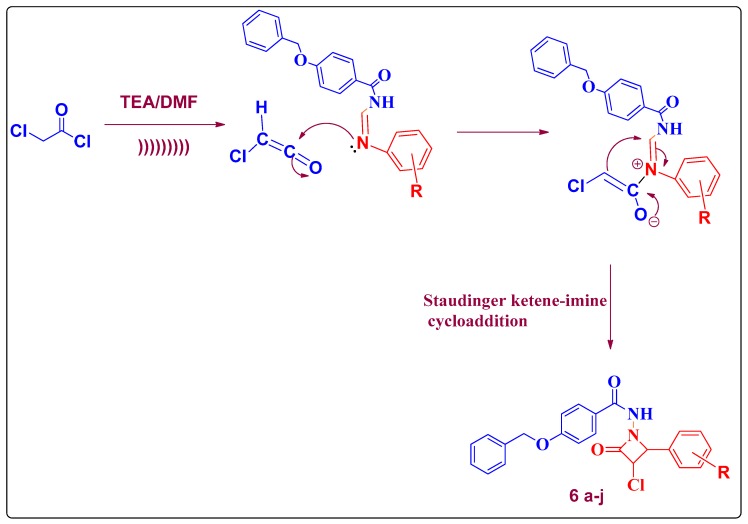
The mechanism of synthesis of 4-(benzyloxy)-*N*-(3-chloro-2-(substituted phenyl)-4-oxoazetidin-1-yl) benzamide **6a**–**j**.

The mechanism of reaction can be explained as shown in Figure 2.

The mechanism of reaction follows the Staudinger ketene-imine cycloaddition reaction. The first step is a nucleophilic attack by the imine nitrogen on the carbonyl carbon (ketene) of chloroacetyl cloride to generate a zwitterionic intermediate. Both the ketene and the imine are molecules that can act as either nucleophiles or electrophiles. The zwitterionic intermediate undergoes stepwise ring closure to give the β-lactam ring.

For the optimization of reaction conditions, the model reaction was carried out by cyclization of intermediate 4-(benzyloxy)-*N*′-(4-hydroxybenzylidene) benzohydrazide with chloro acetyl chloride to give the derivative 4-(benzyloxy)-*N*-(3-chloro-2-(4-hydroxyphenyl)-4-oxoazetidin-1-yl) benzamide **6a**, in various solvents, by using a catalyst with conventional refluxing and ultrasound-assisted modern green techniques. Various solvents were tried and on the basis of yield of the product obtained, dimethyl formamide (DMF) was selected as the solvent for this reaction, which is required in lesser amounts (5 mL). No product was obtained without use of catalyst, hence triethyl amine was selected as the catalyst which gave desired products with a better yield with solvent DMF. The details are as shown in Table 1.

The synthesis of all derivatives of 4-(benzyloxy)-*N*-(3-chloro-2-(substituted phenyl)-4-oxoazetidin-1-yl) benzamide **6a**–**j** was carried out by refluxing and ultrasonic irradiation methods for comparison of conventional and modern green chemistry tools using ultra-sonication. The time required for completion of reaction with yield in percent is mentioned in Table 2.

Herein, we are reporting synthesis of 4-(benzyloxy)-*N*-(3-chloro-2-(substituted phenyl)-4-oxoazetidin-1-yl) benzamide **6a**–**j** derivatives in excellent yield with 78 to 88% by the cyclocondensation of Schiff’s bases **5a**–**j** with chloro acetyl chloride in the presence of the catalyst triethyl amine in solvent DMF by eco-friendly, rapid, and suitable ultrasound-assisted green chemistry protocol. The structures of the synthesized compounds 4-(benzyloxy)-*N*-(3-chloro-2-(substituted phenyl)-4-oxoazetidin-1-yl) benzamide **6a**–**j** were confirmed on the basis of their respective analytical and spectral data like IR, ^1^H-NMR, ^13^C-NMR, mass, and elemental analysis. The spectra of the synthesised compounds are provided in the Supplementary File S2 and the structures and IUPAC names of synthesised derivatives are provided in the Supplementary File S3.

### 2.2. Biological Activity

#### In Vitro Anti-Tubercular Activity

All the synthesized compounds **6a**–**j** were screened for their *in vitro* anti-tubercular activity against MTB H37Ra (ATCC 25177). Rifampicin was used as a positive control. All of the ten synthesized derivatives **6a**–**j** exhibited excellent *in vitro* anti-tubercular activity as shown in Table 3.

Albendazole standard reference drug for docking study, Paclitaxel standard reference drug for cytotoxicity, Rifampicin standard reference drug for anti-tubercular activity, IC_50_ is the inhibition concentration 50% as compared to the growth of control; H37Ra (ATCC 25177) Mycobacterium strain, HeLa Human cervical cancer cell line.

All the synthesized azetidinones derivatives have exhibited promising anti-tubercular activity, which can be assigned to the presence of the benzyloxy group, benzamide group, β-lactam ring and various electron donating, polar groups present on the *para* position and electron withdrawing groups at the *meta* position of the aromatic ring. Derivative **6a** bearing a hydroxyl group at the *para*-position of phenyl exhibited promising anti-tubercular activity (IC_50_ = 0.652 μg/mL). Derivative **6e** bearing nitro as an electron withdrawing group at the *meta*-position of the phenyl ring showed an IC_50_ value of 0.654 μg/mL. Derivative **6g** having a hydroxyl group at the *para*-position and a methoxy group at the *meta*-position of the phenyl ring also showed an IC_50_ value of 0.718 μg/mL. Derivative **6j** with thiophene ring showed an IC_50_ value of 0.786 μg/mL. Derivative **6c** showing a fluoro group at the *para*-position and **6h** with an ethoxy group at the *meta* and a hydroxyl group at the *para* position of the phenyl ring exhibited an IC_50_ value of 0.85 μg/mL. Derivative **6b** containing a methoxy group at the *para* position of the phenyl ring exhibited an IC_50_ value of 0.918 μg/mL. It was observed that bulky substituents reduced the activity; derivative **6i** bearing a benzyloxy group at the *para*-position and derivative **6f** having a di-methoxy group at the *meta* and *para* positions of the phenyl ring exhibited an IC_50_ value of 1.309 and 1.715 μg/mL, respectively. Derivative **6d** showing a chloro group at the *para* position of the phenyl ring exhibited an IC_50_ value of 1.343 μg/mL

### 2.3. In Vitro Cytotoxicity Study

To lowers the failure of potential therapeutics at later stages of clinical trials toxicity analysis of synthesized compounds at the early stage of research work plays a very important role [52]. To examine the safety of the synthesized compounds, **6a**–**j** were evaluated for their toxicity against human cervical cancer cell line HeLa using the concentration range between 100–0.1 μg/mL. The results obtained were summarized in Table 3. The results indicate that all the synthesized compounds **6a**–**j** had an IC_50_ value of more than 30 μg/mL. Hence, the synthesized series could be considered as non-toxic in nature and selective in their anti-mycobacterial activity.

### 2.4. Molecular Docking

Molecular docking studies were carried out in order to understand the binding mode and to identify the structural features of the active derivative from the synthesized series, with the mycobacterial enoyl reductase (InhA) (PDB code 4TZK). In MTB the key enzyme responsible for biosynthesis of cell wall constituents such as mycolic acids is InhA (FabI/ENR), an enoyl-ACP reductase. The wide variety of the agents interfere with synthesis of mycolic acids, essential long alpha-alkylated and β-hydroxylated fatty acids found in the mycobacterial cell wall. InhA is a member of the short chain dehydrogenase reductase superfamily of enzymes. Its attractiveness as a target for the discovery of new antibiotics has been validated some years ago [53,54,55].

**Figure 3:**
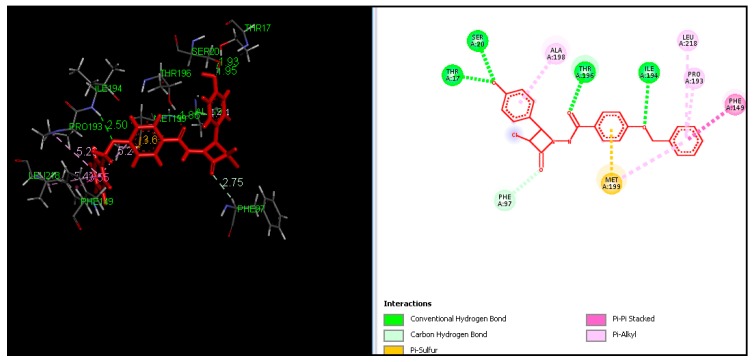
Binding Pose and molecular interactions of 6a into the active site of InhA.

**Figure 4:**
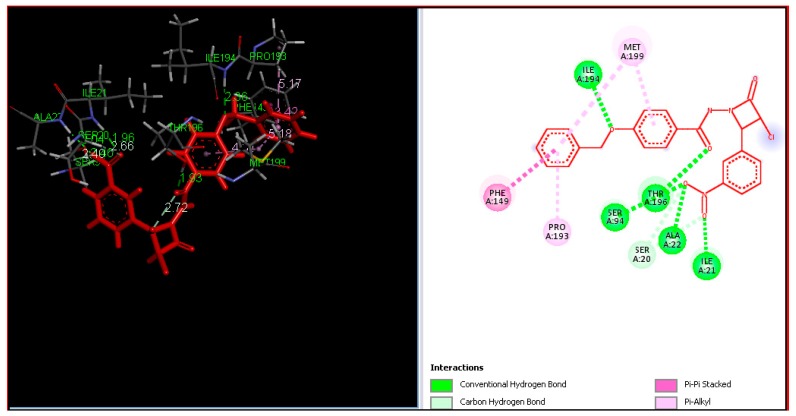
Binding Pose and molecular interactions of 6e into the active site of InhA.

Earlier work by other research groups reported 1,3,4-oxadiazole derivatives to inhibit mycobacterial enoyl reductase (InhA); which gives us the firm basis for selection of InhA as the potential target. The molecular inhibition of enoyl-ACP reductase activity can be well understood by molecular docking study [56,57,58,59], and also helps in correlation of *in vivo* and *in vitro* anti-tubercular activity of azetidin-2-one derivatives. The results of experimental *in vitro* anti-tubercular activity were also replicated in by molecular docking study. All the azetidinone derivatives **6a**–**j** were successfully docked into the active site of target enzyme mycobacterial enoyl reductase (InhA/FabI/ENR) and it was observed that they have varying degrees of affinity to the active site residues. The majority of the amino acids present in the active site cavity such as alanine, isoleucine, isoleucine, threonine, serine, proline, phenylalanine, tyrosine, and methionine are polar and hydrophobic amino acid residues. The detail molecular interactions study in between active site amino acid residue and pharmacophoric features of components of the molecule was carried out to understand the thermodynamic stability of different azetidin-2-one derivatives, and also provide insight information about binding modes observed within the active site cavity. The synthesized derivatives **6a** (8.64), **6e** (8.61), and **6g** (8.47) have shown a high degree of binding affinity towards InhA indicated by total score. The detail analysis of the binding interactions and binding pose of **6a** showed that it is stabilized within the active site of enoyl reductase through an extensive network of favorable non covalent interactions such as conventional hydrogen, carbon bond interaction, and Pi (Pi-sigma, Pi-Sulfur, Pi-Pi stacked interaction and Pi-Alkyl) interactions. The active site polar amino acid THR17 and SER20 forms, conventional hydrogen bond interaction with the oxygen atom of hydroxyl group (–OH) of the phenyl ring with a distance of 1.93 and 1.95 Å, while another polar amino acid THR196 which forms a conventional hydrogen bond interaction with the oxygen atom of (C=O) carbonyl of amide linkage with a distance of 1.86 Å. The hydrophobic amino acid PHE97 interacts with the azetidin-2-one ring carbonyl (C=O) oxygen atom to form carbon hydrogen bond with the distance of 2.75 Å. The ether bridge oxygen atom (–O–) present in between benzyl ring and phenyl ring forms a conventional hydrogen bond interaction with hydrophobic amino acid ILE194 of distance 2.50 Å. The hydrophobic/sulfur containing amino acid MET199 sulfur atom interact with Pi-electron cloud of the bridge ring (phenyl) to form Pi–Sulfur interactions of distance 3.65 Å. The hydrophobic and polar amino acids PHE149, ALA198, MET199, LEU218 and PRO193 interact with Pi electron clouds of aryl rings to form Pi–Pi T stacking and Pi–alkyl interactions shown in Figure 3. The second most active derivative **6e**, having a total score of 8.61, forms a conventional hydrogen bond interaction with active site hydrophobic amino acid ILE21 and ALA22 with nitro group oxygen atoms (O-N=O) of the nitro benzene ring of a distance of 1.96 and 2.04 Å, respectively. Another hydrophobic amino acid ILE194 interacts with the ether bridge oxygen atom (–O–) present between the benzyl ring and phenyl ring forms a conventional hydrogen bond interaction with a distance of 2.36 Å. The polar amino acid THR196 interacts with amide linkage (NH-C=O) to form a conventional hydrogen bond interaction with a distance of 1.93 Å. The polar amino acid SER20 interact with the oxygen atom of the nitrobenzene ring to form a carbon hydrogen bond of distance 2.66 Å. The hydrophobic amino acids PHE149, MET199, and PRO193 interact with Pi electron clouds of aryl rings to form Pi–Pi T stacking, and Pi–alkyl interactions shown in Figure 4.

**Figure 5:**
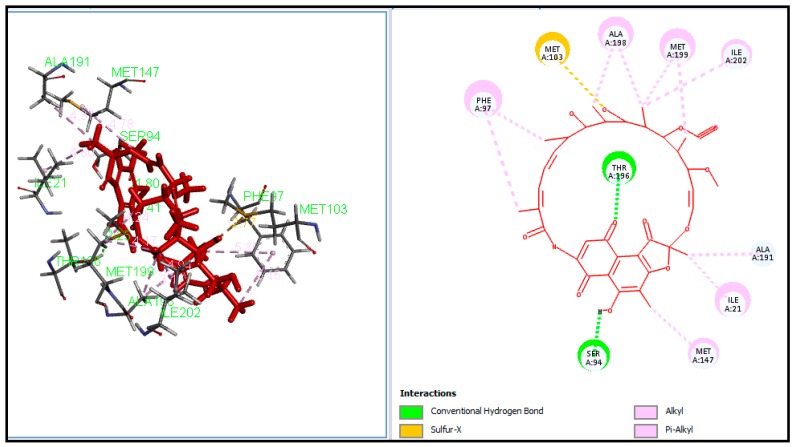
Binding Pose and molecular interactions of Rifampicin into the active site of InhA.

The standard drug used for *in vitro* assay Rifampicin was also docked in the active site of target enzyme mycobacterial enoyl reductase (InhA/FabI/ENR) with a total score of 3.5249. The Polar Amino acids such as SER94 and THR196 form hydrogen bond interactions with the Phenolic hydroxyl group and one of the carboxyl 1,4-dione of distance 1.80 and 2.25 Å respectively. Aliphatic and aromatic hydrophobic amino acids such as PHE97, ALA198, ILE202, ALA191, ILE21, MET147 and 199 form various kind of weak Pi–alkyl and alkyl interactions as shown in Figure 5.

#### Admet Predication

Early prediction of drug-like properties of lead compounds is an important task as it decides the time and cost of drug discovery and development. Many active agents which have shown significant biological activity failed in clinical trials because of inadequate pharmacokinetics drug-like properties [60]. Pharmaceutically relevant properties of synthesized derivatives were calculated and analyzed with various physical descriptors and ADMET prediction by using FAFDrugs2 and data is summarized in Table 4. The drug-like properties have been predicted by analyzing ADME parameters based on Lipinski’s rule of Five and its variants [61]. This approach has been widely used as a filter for substances that are likely to be further developed for drug design programs on the basis of molecular weight (<500), partition coefficient LogP (<5), number of hydrogen bond donors (<10), number of hydrogen bond acceptors (<5), number of rotatable bonds (<10), total polar surface area (<75 Å^2^). The % ABS, percentage absorption, is also predicated using the formula ABS = 109 − (0.345 × TPSA). To increase the rate of drug likeness predication, Veber suggested some variations: molecular weight can be considered below 500 Da, total polar surface area <150 Å^2^, partition coefficient LogP <5.6. The synthesized compounds showed significant values for the various analyzed parameters and showed good drug-like properties based on Lipinski’s rule of five and its variants characterized that these derivatives are likely to be act as orally active agents except derivative **6j** [62,63,64].

The values of %ABS, Polar surface area (PSA), n-HBA and n-HBD for synthesized azetidinone derivatives shows that derivative **6a** to **6i** as compared to **s**tandard drug Albendazole indicated that they have exhibited excellent oral bioavailability as shown in Table 4. The *in silico* assessment of derivative **6a** to **6i** have shown that they posseses very good pharmacokinetic properties which is reflected in their physicochemical values and ultimately contributing to the pharmacological properties of these derivatives.

## 3. Materials and Methods

### 3.1. General Information

All the reactions were performed in oven-dried glassware. All the chemicals used for synthesis were procured from Merck (Mumbai, India), Sigma (Mumbai, India), HiMedia (Mumbai, India), or Qualigens (Mumbai, India) and used without further purification. The ultrasound sonicator (Sonics Vibra-cell, Model no. VCX 500, Sonics & Materials, Inc., Newtown, CT, USA) equipped with a solid synthetic probe, 13 mm in tip diameter, operating at 20 kHz with a maximum power output of 500 W, was used for synthesis of final title compounds. The progress of each reaction was monitored by ascending thin layer chromatography (TLC) using pre-coated silica gel F254 aluminum TLC sheets (Merck, Mumbai, India) and the spots were visualized by UV light and iodine vapors. Elemental analyses (C, H, and N) were done with a FLASHEA 112 Shimadzu’analyzer (Mumbai, India) and all analyses were consistent (within 0.4%) with theoretical values. Infrared (IR) spectra were recorded on a PS 4000 FTIR (JASCO, Tokyo, Japan) using KBr pellets. ^1^H- and ^13^C-NMR (400 MHz) spectra were recorded on an ACF 200 spectrometer (Bruker, Billerica, MA, USA) fitted with an Aspect 3000 computer and all the chemical shifts (ppm) were referred to internal TMS for ^1^H and DMSO-*d*_6_ for ^13^C-NMR. Data for ^1^H-NMR is reported in the order of chemical shift, multiplicity (s, singlet; d, doublet; t, triplet; q, quartet; br, broad; br s, broad singlet; m, multiplet and/or multiple resonance), number of protons. A Micro TOF-Q-II (Bruker Daltonics, Billerica, MA, USA) with electron spray ionization (ESI) was used to obtain the HRMS data.

(a) General procedure for the synthesis of 4-(benzyloxy) benzoate (**3**) [51]:

Synthesis of methyl-4-(benzyloxy) benzoate was carried out in an ultrasonic processor by taking the equivalent ratio of benzyl chloride and methyl-4-hydroxybenzoate (0.01 mol) using DMF as solvent in a beaker with a catalytic amount of K_2_CO_3_. Reaction was completed in four hours. The completion of reaction was monitored by TLC. The reaction mixture was poured into ice-water. The product obtained was filtered, dried, and recrystallized from ethanol. Colour: White M.P.105 °C.

(b) General procedure for the synthesis of 4-(benzyloxy) benzo hydrazide (**4**):

Synthesis of substituted benzo hydrazide was carried out in an ultrasonic processor by taking the equivalent ratio of a mixture of corresponding esters (20 mmol) and 85% hydrazine hydrate (20 mmol) in ethanol for three hours. The completion of reaction was monitored by TLC. The reaction mixture was poured into ice-water. The solid obtained was filtered, dried, and recrystallized from ethanol. Colour: White, M.P.: 80 °C.

(c) General procedure for the synthesis of 4-(benzyloxy)-*N*′-(substituted benzylidene) benzo hydrazide, called Schiff’s bases **5a**–**j**:

Schiff’s bases **5a**–**j** were obtained by condensation of equimolar quantities of 4-(benzyloxy) benzo hydrazide (0.01 mol) and different substituted aldehydes (0.01 mol) in the presence of glacial acetic acid (0.02 mol) as a catalyst in absolute ethanol kept in an ultra-sonicator for 1 to 2 h. The completion of the reaction was monitored by TLC. The reaction mixture was concentrated and cooled. The obtained solid was filtered and dried. The product was recrystallized from ethanol.

(d) General method of synthesis of 4-(benzyloxy)-*N*-(3-chloro-2-(substituted phenyl)-4-oxoazetidin-1-yl) benzamide **6a**–**j**:

In a borosil beaker, a mixture of Schiff’s base (0.01 mol), chloroacetyl chloride (0.02 mol), and triethyl amine (0.02 mol) as a catalyst was dissolved in DMF (5 mL) and the beaker was kept in an acoustic chamber, the solid probe of ultrasound was lowered down in the beaker so as to be immersed in the solvent and subjected to ultra-sonication at room temperature for 2 to 2.30 h. The completion of the reaction was monitored by TLC. The resulting solid was filtered, washed several times with water, dried, and then recrystallized from ethanol. The melting point and yield were recorded.

Structures of the synthesized derivatives **6a**–**j** were confirmed by spectral studies as reported below:

*4-(Benzyloxy)-N-(3-chloro-2-(4-hydroxyphenyl)-4-oxoazetidin-1-yl) benzamide* (**6a**), IR (KBr) ν_max_ (cm^−1^): 3497.24 OH stretching, 3430 NH stretching, 3108–3009 aromatic CH stretching, 2880–2870 aliphatic CH stretching, 1630.56 C=O stretching of amide; ^1^H-NMR (DMSO-*d*_6_), δ ppm: 4.92 (d, 1H, *J* = 8 Hz, –CH–), 5.15 (s, 2H, –CH_2_–), 5.50 (s, 1H, –OH), 5.80 (d, 1H, *J* = 8 Hz, –CH–), 6.79–8.34 (m, 5H, 4H, 4H of three aromatic rings), 8.46 (s, 1H of NH); ^13^C-NMR (DMSO-*d*_6_), δ ppm: 64.1, 67.4, 69.4, 114.0(2), 116.1(2), 124.4, 127.0(2), 127.2(2), 127.6, 128.1(2), 129.2(2), 136.1(2), 157.2, 161.0, 164.9 and 165.1; MS (ESI) *m/z*: 424.41 [M + 2]^+^; Molecular Formula: C_23_H_19_ClN_2_O_4_; Elemental Analysis: Calculated (C, H, Cl, N) 65.33, 4.53, 8.38, 6.62. Found: 65.30, 4.51, 8.36, 6.64.

*4-(Benzyloxy)-N-(3-chloro-2-(4-methoxyphenyl)-4-oxoazetidin-1-yl) benzamide* (**6b**), IR (KBr) ν_max_ (cm^−1^): 3335.41 NH stretching, 3152.77 CH stretching of aromatics, 2876.58 CH stretching of alkyl, 1640.56 C=O stretching of amide, 1215. 22 C–O stretching of –OCH_3_; ^1^H-NMR (DMSO-*d*_6_), δ ppm: 3.79 (s, 3H, –OCH_3_), 5.03 (d, 1H, *J* = 8 Hz, –CH–), 5.10 (s, 2H, –CH_2_–), 5.49 (d, 1H, *J* = 8 Hz, –CH–), 6.99–7.92 (m, 5H, 4H, 4H of three aromatic rings), 8.4 (s, 1H of NH); ^13^C-NMR (DMSO-*d*_6_), δ ppm: 54.8, 63.1, 66.4, 74.8, 109.1(2), 111.4(2), 121.3, 124.6(2), 126.1(2), 128.6, 129.5(2), 132.9(2), 134.8, 135.7, 155.6, 158.4, 161.5 and 162.9; MS (ESI) *m/z:* 438.12 [M + 2]^+^; Molecular Formula: C_24_H_21_ClN_2_O_4_; Elemental Analysis: Calculated (C, H, Cl, N) 65.98, 4.84, 8.11, 6.41. Found: 65.95, 4.87, 8.13, 6.39.

*4-(Benzyloxy)-N-(3-chloro-2-(4-fluorophenyl)-4-oxoazetidin-1-yl) benzamide* (**6c**), IR (KBr) ν_max_ (cm^−1^): 3530 NH stretching, 3160–3080 aromatic CH stretching, 2879–2819 aliphatic CH stretching, 1670.56 C=O stretching of amide, 1260 C–F stretching; ^1^H-NMR (DMSO-*d*_6_), δ ppm: 5.05 (d, 1H, *J* = 8 Hz, –CH–), 5.19 (s, 2H, –CH_2_–), 5.51 (d, 1H, *J* = 10 Hz, –CH–), 7.02–7.97 (m, 5H, 4H, 4H of three aromatic rings), 8.6 (s, 1H of NH); ^13^C-NMR (DMSO-*d*_6_), δ ppm: 61.1, 63.4, 75.8, 108.4(2), 112.3(2), 126.3, 128.1(2), 130.6, 132.5(4), 135.9(2), 138.7, 143.1, 158.9, 160.4, 161.5 and 162.0; MS (ESI) *m/z:* 428.10 [M + 4]^+^; Molecular Formula: C_23_H_18_ClFN_2_O_3_; Elemental Analysis: Calculated (C, H, Cl, F, N) 65.02, 4.27, 8.34, 4.47, 6.59. Found: 65.05, 4.25, 8.33, 4.49, 6.57.

*4-(Benzyloxy)-N-(3-chloro-2-(4-chlorophenyl)-4-oxoazetidin-1-yl) benzamide* (**6d**), IR (KBr) ν_max_ (cm^−1^): 3590 NH stretching, 3177–3050 aromatic CH stretching, 2880–2890 aliphatic CH stretching, 1670.56 C=O stretching of amide, 744 C–Cl stretching; ^1^H-NMR (DMSO-*d*_6_), δ ppm: 5.03 (d, 1H, *J* = 10 Hz, –CH–), 5.12 (s, 2H, –CH_2_–), 5.49 (d, 1H, *J* = 8 Hz, –CH–), 7.10–7.88 (m, 5H, 4H, 4H of three aromatic rings), 8.3 (s, 1H of NH); ^13^C-NMR (DMSO-*d*_6_), δ ppm: 55.1, 57.4, 60.8, 108.4(2), 116.3, 118.1(2), 122.2(2), 125.6, 126.5(2), 127.6(2), 128.6(2), 131.3, 135.7, 146.6, 160.4, 161.5 and 164.7; MS (ESI) *m/z:* 445.07 [M + 4]^+^. Molecular Formula: C_23_H_18_Cl_2_N_2_O_3_; Elemental Analysis: Calculated (C, H, Cl, N) 62.60, 4.11, 16.07, 6.35. Found: 62.63, 4.09, 16.06, 6.33.

*4-(Benzyloxy)-N-(3-chloro-2-(3-nitrophenyl)-4-oxoazetidin-1-yl) benzamide* (**6e**), IR (KBr) ν_max_ (cm^−1^): 3520 NH stretching, 3150–3040 aromatic CH stretching, 2877–2880 aliphatic CH stretching, 1680.56 C=O stretching of amide, 1345 NO_2_ stretching; ^1^H-NMR (DMSO-*d*_6_), δ ppm: 5.04 (d, 1H, *J* = 10 Hz, –CH–), 5.15 (s, 2H, –CH_2_–), 5.56 (d, 1H, *J* = 8 Hz, –CH–), 6.80–8.23 (m, 5H, 4H, 4H of three aromatic rings), 8.43 (s, 1H of NH); ^13^C-NMR (DMSO-*d*_6_), δ ppm: 64.2, 67.6, 69.1, 114.0(2), 116.1(2), 124.4, 127.0(2), 127.2(2), 127.6, 128.2(2), 129.1(2), 136.0(2), 157.2, 161.9, 164.1 and 165.0; MS (ESI) *m*/*z*: 453.18 [M + 2]^+^; Molecular Formula: C_23_H_18_ClN_3_O_5_; Elemental Analysis: Calculated (C, H, Cl, N) 61.14, 4.02, 7.85, 9.30. Found: 61.16, 4.03, 7.88, 9.27.

*4-(Benzyloxy)-N-(3-chloro-2-(3,4-dimethoxyphenyl)-4-oxoazetidin-1-yl) benzamide* (**6f**), IR (KBr) ν_max_ (cm^−1^): 3590 NH stretching, 3166–3044 aromatic CH stretching, 2880–2820 aliphatic CH stretching, 1649.56 C=O stretching of amide, 1400 C–OCH_3_; ^1^H-NMR (DMSO-*d*_6_), δ ppm: 3.85 (s, 6H, –OCH_3_), 5.10 (d, 1H, *J* = 8 Hz, –CH–), 5.22 (s, 2H, –CH_2_–), 5.47 (d, 1H, *J* = 8 Hz, –CH–), 6.70–7.89 (m, 5H, 4H, 3H of three aromatic rings), 8.7 (s, 1H of NH); ^13^C-NMR (DMSO-*d*_6_), δ ppm: 52.1(2), 60.1, 64.7, 68.8, 105.8, 110.4(2), 113.9, 116.9, 118.3, 125.1(2), 126.6, 132.5(2), 134.9(2), 138.7, 140.8, 145.8, 152.6, 158.4, 159.5 and 160.7; MS (ESI) *m/z*: 468.13 [M + 2]^+^. Molecular Formula: C_25_H_23_ClN_2_O_5_; Elemental Analysis: Calculated (C, H, Cl, N) 64.31, 4.97, 7.59, 6.00. Found: 64.32, 4.95, 7.60, 6.03.

*4-(Benzyloxy)-N-(3-chloro-2-(4-hydroxy-3-methoxyphenyl)-4-oxoazetidin-1-yl) benzamide* (**6g**), IR (KBr) ν_max_ (cm^−1^): 3500 OH stretching, 3480 NH stretching, 3100–3070 aromatic CH stretching, 2880–2830 aliphatic CH stretching, 1670.56 C=O stretching of amide,1440 C–OCH_3_; ^1^H-NMR (DMSO-*d*_6_), δ ppm: 3.79 (s, 3H, –OCH_3_), 5.12 (d, 1H, *J* = 6 Hz, –CH–), 5.21 (s, 2H, –CH_2_–), 5.39 (s, 1H, –OH), 5.48 (d, 1H, *J* = 4 Hz, –CH–), 6.58–7.97 (m, 5H, 4H, 3H of three aromatic rings), 8.6 (s, 1H of NH); ^13^C-NMR (DMSO-*d*_6_), δ ppm: 50.1, 61.1, 65.7, 73.8, 108.2, 112.4(2), 113.4, 115.3, 122.3, 125.1(2), 126.6, 127.5(2), 129.9(2), 135.7, 139.1, 148.7, 149.3, 159.4, 161.5 and 162.9; MS (ESI) *m/z*: 454.11 [M + 2]^+^; Molecular Formula: C_24_H_21_ClN_2_O_5_; Elemental Analysis: Calculated (C, H, Cl, N) 63.65, 4.67, 7.83, 6.19. Found: 63.62, 4.65, 7.80, 6.21.

*4-(Benzyloxy)-N-(3-chloro-2-(3-ethoxy-4-hydroxyphenyl)-4-oxoazetidin-1-yl) benzamide* (**6h**), IR (KBr) ν_max_ (cm^−1^): 3530 OH stretching, 3470 NH stretching, 3160–3087 aromatic CH stretch, 2890–2890, aliphatic CH stretching, 1690.56 C=O stretching of amide,1440 stretching C–OCH_3_; ^1^H-NMR (DMSO-*d*_6_), δ ppm: 1.27 (t, 3H, –OCH_3_), 4.02 (q, 2H, –CH_2_–), 5.09 (d, 1H, *J* = 8 Hz, –CH–), 5.21 (s, 2H, –CH_2_–), 5.42 (s, 1H, –OH), 5.52 (d, 1H, –CH–), 6.61–7.99 (m, 5H, 4H, 3H of three aromatic rings), 8.3 (s, 1H of NH); ^13^C-NMR (DMSO-*d*_6_), δ ppm: 10.8, 61.1, 63.9, 65.7, 75.8, 115.3, 116.4(2), 118.0, 119.6, 123.3, 125.1(2), 126.6, 132.5(2), 133.9(2), 135.7(2), 147.8, 150.0, 155.5, 157.5 and 160.7 MS (ESI) *m*/*z*: 468.13 [M + 2]^+^; Molecular Formula: C_25_H_23_ClN_2_O_5_; Elemental Analysis: Calculated (C, H, Cl, N) 64.31, 4.97, 7.59, 6.00. Found: 64.36, 4.95, 7.56, 6.02.

*4-(Benzyloxy)-N-(2-(4-(benzyloxy) phenyl)-3-chloro-4-oxoazetidin-1-yl) benzamide* (**6i**), IR (KBr) ν_max_ (cm^−1^): 3480 NH stretching, 3170–3080 aromatic CH stretching, 2879–2833 aliphatic CH stretching, 1648.56 C=O stretching of amide; ^1^H-NMR (DMSO-*d*_6_), δ ppm: 5.05 (d, 1H, *J* = 8 Hz, –CH–), 5.10 (s, 4H, –CH_2_–), 5.49 (d, 1H, *J* = 8 Hz, –CH–), 6.90–7.95 (m, 5H, 4H, 4H, 5H of four aromatic rings), 8.6 (s, 1H of NH); ^13^C-NMR (DMSO-*d*_6_), δ ppm: 60.1, 66.4, 75.8(2), 110.1(2), 111.5(2), 125.3, 127.6(2), 128.1(4), 129.6(2), 132.5(2), 132.9(4), 134.8, 137.7(2), 158.0, 161.4, 164.5 and 165.7; MS (ESI) *m/z:* 514.15 [M + 2]^+^. Molecular Formula: C_30_H_25_ClN_2_O_4_; Elemental Analysis: Calculated (C, H, Cl, N) 70.24, 4.91, 6.91, 5.46. Found: 70.22, 4.89, 6.93, 5.44.

*4-(Benzyloxy)-N-(3-chloro-2-oxo-4-(thiophen-2-yl) azetidin-1-yl) benzamide* (**6j**), IR (KBr) ν_max_(cm^−1^): 3530 NH stretching, 3100–3010 aromatic CH stretching, 2880–2860 aliphatic CH stretching, 1660.56 C=O stretching of amide; ^1^H-NMR (DMSO-*d*_6_), δ ppm: 5.02 (d, 1H, *J* = 8 Hz, –CH–), 5.11 (s, 2H, –CH–), 5.51 (d, 1H, *J* = 12 Hz, –CH–), 6.77 (d, 1H, 3rd –CH– of Thiophene), 6.89 (t, 1H, 4th –CH– of Thiophene), 7.47 (m, 1H, 5th –CH– of Thiophene), 7.11–8.0 (m, 5H, 4H, aromatic rings), 8.4 (s, 1H of NH); ^13^C-NMR (DMSO-*d*_6_), δ ppm: 61.6, 62.8, 71.8, 106.4(2), 115.3, 118.0, 121.1(2), 124.6, 128.7, 131.0, 132.5(2), 134.9(2), 137.3, 139.7, 158.4, 159.5 and 161.1; MS (ESI) *m/z:* 414.06 [M + 2]^+^. Molecular Formula: C_21_H_17_ClN_2_O_3_S; Elemental Analysis: Calculated (C, H, Cl, N, S) 61.09, 4.15, 8.59, 6.78, 7.77. Found: 63.97, 3.65, 8.61, 7.94, 7.74.

### 3.2. Biological Activity

*In Vitro* Anti-Tubercular Activity by the XTT Reduction Menadione Assay (XRMA) Method

Mycobacterium tuberculosis H37Ra (ATCC 25177) strains were obtained from AstraZeneca, India. The stock culture was maintained at −80 °C and subcultured once in a liquid M. pheli medium which contained 0.5 g KH_2_PO_4_, 0.25 g trisodium citrate, 60 mg MgSO_4_, 0.5 gm asparagine, and 2 mL glycerol in distilled water (100 mL) followed by pH adjustment to 6.6. Bacilli stock cultures were grown in M. pheli medium at 37 °C with continuous agitation at 150 RPM;, it takes at least 8–10 days for OD 1 at 620 nm. Stock solutions (10 mg/mL) of all the synthesized compounds were prepared in DMSO and were evaluated for *in vitro* anti-tubercular activity against *Mycobacterium tuberculosis* H37Ra (ATCC 25177) in a liquid medium using an established XTT Reduction Menadione assay (XRMA) method. Briefly, the 96 wells plate received 248.5 μL of M. Pheli medium consisting of bacilli and 2.5 μL of test solutions in serial dilution (12.5, 6.25, 3.125, 1.5625, 0.78 μg/mL). The plates were sealed with a 96-well sealing sheet and incubated at 37 °C for 8 days. Post incubation, XRMA was carried out to estimate viable cells present in different wells of the assay plate [65]. The absorbance was read on a micro plate reader (Spectramax plus384 plate reader, Molecular Devices Inc., San Jose, CA, USA) at 470 nm filter against a blank prepared from cell-free wells. Absorbance given by cells treated with the vehicle alone was taken as 100% cell growth. MIC and IC_50_ values of compounds were calculated by using Origin 6 software. Percent inhibition was calculated by using following formula:

% Inhibition = [(absorbance of Control − absorbance of Test)/(absorbance of Control − absorbance of Blank)] × 100(1)

where control is the medium with bacilli along with vehicle and blank is cell-free medium. The drug in clinical use, rifampicin was used as a reference.

### 3.3. In Vitro Cytotoxicity Studies by MTT Assay

All the synthesized compounds were tested for their cytotoxicity by a modified MTT assay as described previously [66]. Briefly, cells were seeded at the density of 1 × 10^5^ cells/mL in a 96-well plate. The plates were incubated overnight in CO_2_ incubator (37 °C under 5% CO_2_ and 95% air in a humidified atmosphere). Next day, cells were treated with synthesized compounds at 10-fold dilution (100–0.1 µg/mL) and incubated for an additional 48 h. Paclitaxel was used as positive control. Post incubation, cell medium was replaced with MTT (0.5 mg/mL)-PBS medium and incubated for 2–4 h to form the reduced MTT or Formazan crystals. This reduced MTT or Formazan crystals were solubilized by addition of 100 µL of SDS-DMF (20% SDS in 50% DMF). The optical density was read on a microplate reader (Spectramax plus 384 plate reader, Molecular Devices Inc.) at 570 nm filter against a blank prepared from cell-free wells. Absorbance given by cells treated with the vehicle alone was taken as 100% cell growth. IC_50_ and MIC values were calculated from graphs, using Origin Pro software. Where DMSO treated culture cells is control and culture medium without cells is blank. The percent cytotoxicity in the presence of test fractions was calculated by the following formula:

Percent cytotoxicity = [(average absorbance of control − absorbance of a compound)/(absorbance of control − absorbance of blank)] × 100(2)

### 3.4. Molecular Docking

Molecular Docking study was performed with Tripos SYBYL X 2. 2.1 program [67]. The complex X-ray crystal structures of enoyl-ACP reductase with inhibitors pyrrolidine carboxamide (PDC) for *Mycobacterium* was retrieved from the RCSB protein data bank (http://www.rcsb.org/pdb) and used for the docking study. All the derivatives **6a**–**j** were successfully docked into the active site of target enzyme mycobacterial enoyl reductase (InhA/FabI/ENR) and it was observed that they have varying degrees of affinity to the active site residues.

### 3.5. In Silico ADMET Prediction

Computational study of the synthesized compounds **6a**–**j** was performed for prediction of ADMET properties. The absorption, distribution, metabolism, excretion, and toxicity (ADMET) properties of all the compounds were predicted using software FAFDrugs2. In the present study, molecular weight (M.W.) was calculated. Octanol–water partition coefficient (log Po/w), number of hydrogen bond acceptors (n-ON), number of hydrogen bonds donors (n-OHNH), percentage human oral absorption (% ABS), and Van der Waals surface area of polar nitrogen and oxygen atoms (Polar Surface Area) were also predicted.

## 4. Conclusions

In this research work, we have developed an ecofriendly and efficient ultrasound assisted protocol for synthesis of *4-(benzyloxy)-N-(3-chloro-2-(substituted phenyl)-4-oxoazetidin-1-yl) benzamide* **6a**–**j**. The remarkable benefits of ultra-sonication as a green synthetic strategy are as follows: (1) reactions were carried at room temperature; (2) it required much less time for completion of the reaction as compared to conventional refluxing, hence the ultrasound methodology saves time and electricity; (3) a highly accelerated reaction rate; (4) the use of a much lesser amount of solvent DMF; (5) a shortened and clean work-up procedure; and (6) it is ecofriendly as the reactions are carried out in closed acoustic chamber. The structure analogy of the reported azetidinone derivatives with a β-lactam ring enhance the potential of *4-(benzyloxy)-N-(3-chloro-2-(substituted phenyl)-4-oxoazetidin-1-yl) benzamide* to be developed as antimychobacterial agents and can act as a promising scaffold for lead optimization and drug discovery. Computational molecular docking study demonstrated that **6a**, **6e**, and **6g** are the most active amongst the synthesized derivatives and have the potential for anti-mycobacterial action, supporting the experimental anti-tubercular activity results. Compound **6a** showed promising *in vitro* anti-tubercular activity against Mycobacterium tuberculosis H37Ra (ATCC 25177) strains with IC_50_ 0.652 μg/mL, also proved by the docking score of 8.647 which shows that it inhibits enzyme InhA (FabI/ENR). Compound **6e** showed promising *in vitro* anti-tubercular activity with IC_50_ 0.654 μg/mL, similarly it also inhibits enzyme InhA (FabI/ENR) with a docking score of 8.6156. Compound **6g** also showed good *in vitro* anti-tubercular activity with IC_50_ 0.718 μg/mL, similarly it also inhibits enzyme InhA (FabI/ENR) with a docking score of 8.476. In conclusion, compounds **6a**, **6e**, and **6g** not only give good docking scores, but also show inhibition concentration 50% (IC_50_) anti-tubercular activity. As compared to the standard drug Rifampicin, these derivatives were found to be the competent moieties with potent anti-tubercular activity. The pharmacokinetics/drug like properties of synthesized derivative provide a potential pharmacophore for further drug development process. Thus, the results of the molecular docking study and anti-tubercular activity data of these 4-(benzyloxy)-*N*-(3-chloro-2-(substituted phenyl)-4-oxoazetidin-1-yl) benzamide **6a**–**j** proves that these compounds have the potential to be developed as lead anticancer molecules and there are new opportunities for possible modification in the synthesized series, as per the pharmaceutical requirement in future.

## Figures and Tables

**Table 1:**
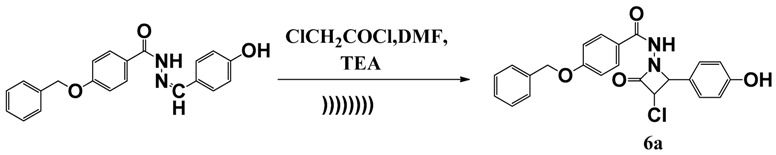
Optimization of reaction conditions for 4-(benzyloxy)-*N*-(3-chloro-2-(4-hydroxyphenyl)-4-oxoazetidin-1-yl) benzamide **6a** derivative using various solvents with and without the use of a catalyst.

Entry	Catalyst	Solvent	Method A Conventional Reflux		Method B Ultrasound Assisted	
			Time (h)	Yield (%)	Time (h)	Yield (%)
1	No Catalyst	Benzene	22	-	6	-
2	No Catalyst	1,4-Dioxane	15	-	6	-
3	No Catalyst	DMF	12	-	5	-
4	Triethyl amine(TEA)	Benzene	16	55	4	65
5	Triethyl amine(TEA)	1,4-Dioxane	12	60	3	75
6	Triethyl amine(TEA)	DMF	08	70	2	88

**Table 2:** Comparison of reaction kinetics of conventional refluxing and ultrasonic irradiation methods for the synthesized compounds **6a**–**j**.

Entry	Conventional Refluxing		Ultrasonic Irradiation	
	Time (h)	Yield (%)	Time (h)	Yield (%)
**6a**	08.00	70	02.00	88
**6b**	06.00	65	02.00	82
**6c**	06.50	72	02.10	80
**6d**	07.00	70	02.20	79
**6e**	07.50	71	02.15	80
**6f**	08.00	65	02.00	79
**6g**	07.50	67	02.00	86
**6h**	07.50	61	02.30	81
**6i**	08.00	66	02.00	78
**6j**	08.00	65	02.00	79

**Table 3:** *In-vitro* anti-tubercular activity, cytotoxicity, and molecular docking of synthesized derivatives **6a**–**j**.

Sr. No	*Mycobacterium tuberculosis* H37Ra (ATCC 25177) IC_50_ (μg/mL)	Hela Cells IC_50_ μg/mL	Molecular Docking Score
			Total Score against 4TZK (-log Ki)
**6a**	0.652	82.62	8.647
**6b**	0.918	54.855	6.9851
**6c**	0.85	31.612	7.5334
**6d**	1.343	70.36	6.9795
**6e**	0.654	>100	8.6156
**6f**	1.715	44.087	6.34
**6g**	0.718	>100	8.476
**6h**	0.85	>100	7.082
**6i**	1.309	89.187	6.9839
**6j**	0.786	33.182	8.0345
**Albendazole**	NA	NA	6.073
**Paclitaxel**	NA	0.0056	NA
**Rifampicin**	0.004	NA	NA

**Table 4:** The Pharmacokinetic parameters important for agents to have excellent oral bioavailability of synthesized compounds.

ID	MW	% ABS	LogP	PSA	Rot. B	RigidB	HBD	HBA	Ratio H/C	Toxicity
**6a**	422.861	81.7899	4.1357	78.87	6	25	2	4	0.304	Non Toxic
**6b**	436.888	85.5849	4.4387	67.87	7	25	1	4	0.291	Non Toxic
**6c**	424.852	88.7692	4.5692	58.64	6	25	1	3	0.304	Non Toxic
**6d**	441.307	88.7692	5.0835	58.64	6	25	1	3	0.304	Non Toxic
**6e**	451.867	73.3477	4.7529	103.34	7	26	2	5	0.391	Non Toxic
**6f**	466.914	82.4005	4.4473	77.1	8	25	1	5	0.32	Non Toxic
**6g**	452.887	78.6055	4.1443	88.1	7	25	2	5	0.333	Non Toxic
**6h**	466.914	78.6055	4.5344	88.1	8	25	2	5	0.32	Non Toxic
**6i**	512.984	85.5849	6.0091	67.87	9	31	1	4	0.233	Non Toxic
**6j**	412.889	79.0264	4.4916	86.88	6	24	1	4	0.333	Toxic
**Albendazole**	281.331	73.365	3.1975	103.29	5	13	2	4	0.583	Non Toxic

MW: molecular weight, LogP: logarithm of partition coefficient of compound between n-octanol and water, PSA: Polar surface area, n-RotBond: number of rotatable bonds, HBA: hydrogen bond acceptors and HBD: hydrogen bond donor.

## Supplementary Materials

The following are available online at http://www.mdpi.com/1420-3049/23/8/1945/s1, Table S1: Physical characterization, S2: Spectral Data, S3: Compound structures with IUPAC names.
