# Supplementary material for: Ultrasound Assisted Synthesis of 4-(Benzyloxy)-N-(3-chloro-2-(substitutedphenyl)-4-oxoazetidin-1-yl) Benzamide as Challenging Anti-Tubercular Scaffold
Source: Molecules. 2018 Aug 3;23(8):1945. doi: 10.3390/molecules23081945 (PMC6222352; doi:10.3390/molecules23081945)
Supplement: Supplementary file 1 [file molecules-23-01945-s001.pdf]

Ultrasound assisted synthesis of 4-(benzyloxy)-N-(3-chloro-2-(substitutedphenyl)-4-oxoazetidin-1-yl) benzamide as challenging anti tubercular scaffold

Urja D. Nimbalkar[1], Julio A. Seijas[2], Rachna Borkute[3], Manoj G. Damale [4], Jaiprakash N. Sangshetti[5], Dhiman Sarkar [3], Anna Pratima G. Nikalje*[5]

[1] Maulana Azad P. G. and Research Centre, Dr. Rafiq Zakaria Campus, Rauza Baug, Aurangabad 431001, India ; urjasatish@gmail.com

[2] Departamento de Química Orgánica, Facultad de Ciencias, Universidad of Santiago de Compostela, Alfonso X el Sabio, 27002 Lugo, Spain; julioa.seijas@usc.es

[3]Combichem-Bio Resource Centre, Division of Organic Chemistry, CSIR-National Chemical Laboratory, Pune 411008 , Maharashtra, India; rachnabrkt@gmail.com ; d.sarkar@ncl.res.in

[4] Shreeyash College of Pharmacy, Aurangabad 431116, India; pharmalink1985@gmail.com

[5] Department of Pharmaceutical Chemistry, Dr. Rafiq Zakaria Campus, Y. B. Chavan College of Pharmacy, Aurangabad 431001, M.S. India ; jnsangshetti@rediffmail.com

* Correspondence: annapratimanikalje@gmail.com ; Tel.: +91-9168929111

Table S1: Physical characterization of 4-(benzyloxy)-N-(3-chloro-2-(substituted phenyl)-4-oxoazetidin-1-yl) benzamide **6a-j**

Code No.	Structure	Molecular Formula	Mol. Wt.	Yield (%)	M.P.(˚C)
**6a**	4-Hydroxyphenyl	$C_{23}H_{19}ClN_2O_4$	422.86	88	175
**6b**	4-Methoxyphenyl	$C_{24}H_{21}ClN_2O_4$	436.89	82	170
**6c**	4-Fluorophenyl	$C_{23}H_{18}ClFN_2O_3$	424.85	80	144
**6d**	4-Chlorophenyl	$C_{23}H_{18}Cl_2N_2O_3$	441.31	79	152
**6e**	3-Nitrophenyl	$C_{23}H_{18}ClN_3O_5$	451.86	80	198
**6f**	3,4-Dimethoxyhenyl	$C_{25}H_{23}ClN_2O_5$	466.91	79	182
**6g**	4-Hydroxy-3-methoxyphenyl	$C_{24}H_{21}ClN_2O_5$	452.89	86	164
**6h**	3-Ethoxy-4-hydroxyphenyl	$C_{25}H_{23}ClN_2O_5$	466.91	81	156
**6i**	4-Benzyloxyphenyl	$C_{30}H_{25}ClN_2O_4$	512.98	78	180
**6j**	Thiophen-2-yl	$C_{21}H_{17}ClN_2O_3S$	412.89	79	172

S2: Mass, ^{1}HNMR and ^{13}C NMR and elemental analysis of representative derivatives.

Mass Spectra: 6a) ***4-(benzyloxy)-N-(3-chloro-2-(4-hydroxyphenyl)-4-oxoazetidin-1-yl) benzamide:***

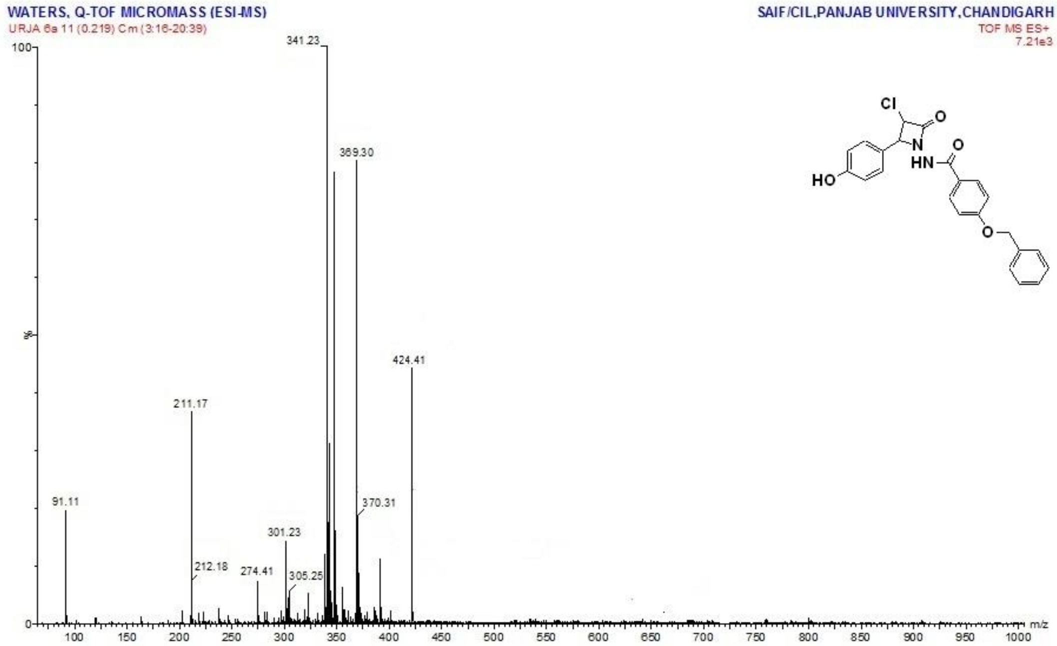

^{1}H NMR Spectra: 6a) ***4-(benzyloxy)-N-(3-chloro-2-(4-hydroxyphenyl)-4-oxoazetidin-1-yl) benzamide:***

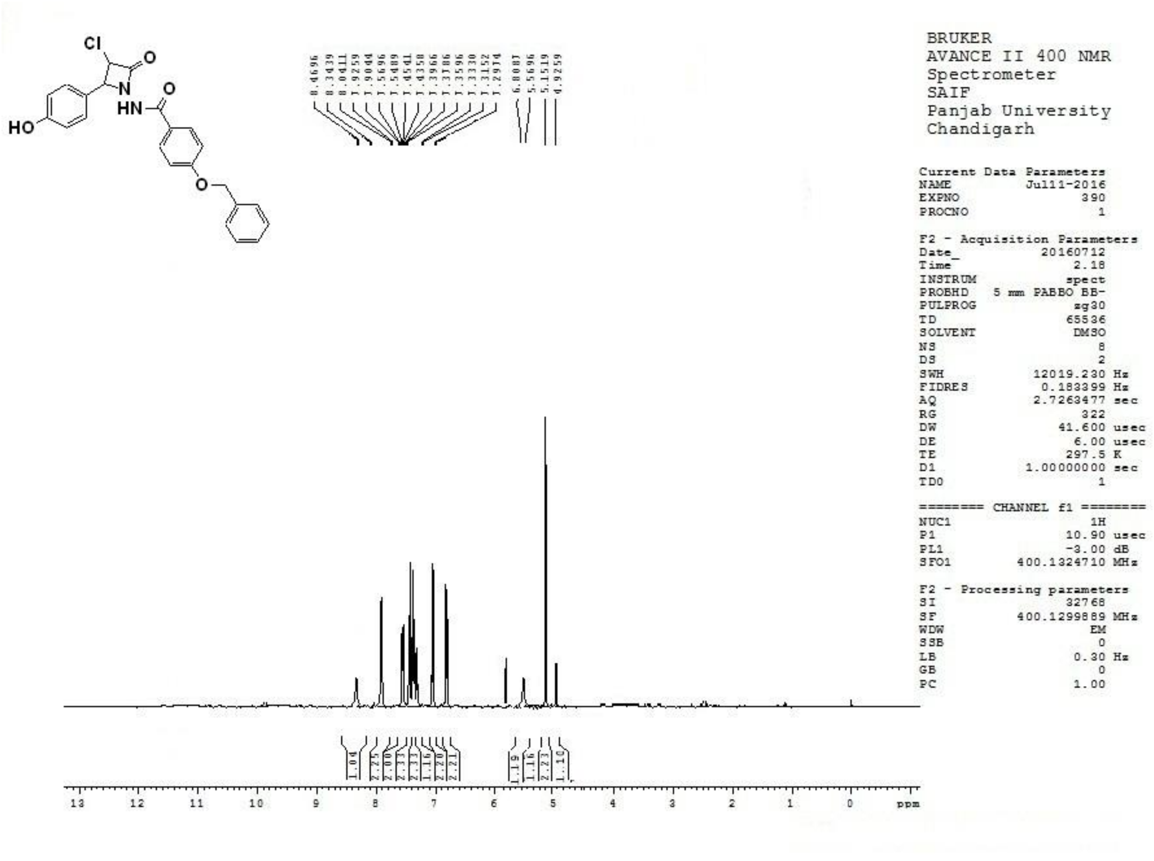

^{13}C NMR Spectra: 6a) ***4-(benzyloxy)-N-(3-chloro-2-(4-hydroxyphenyl)-4-oxoazetidin-1-yl) benzamide:***

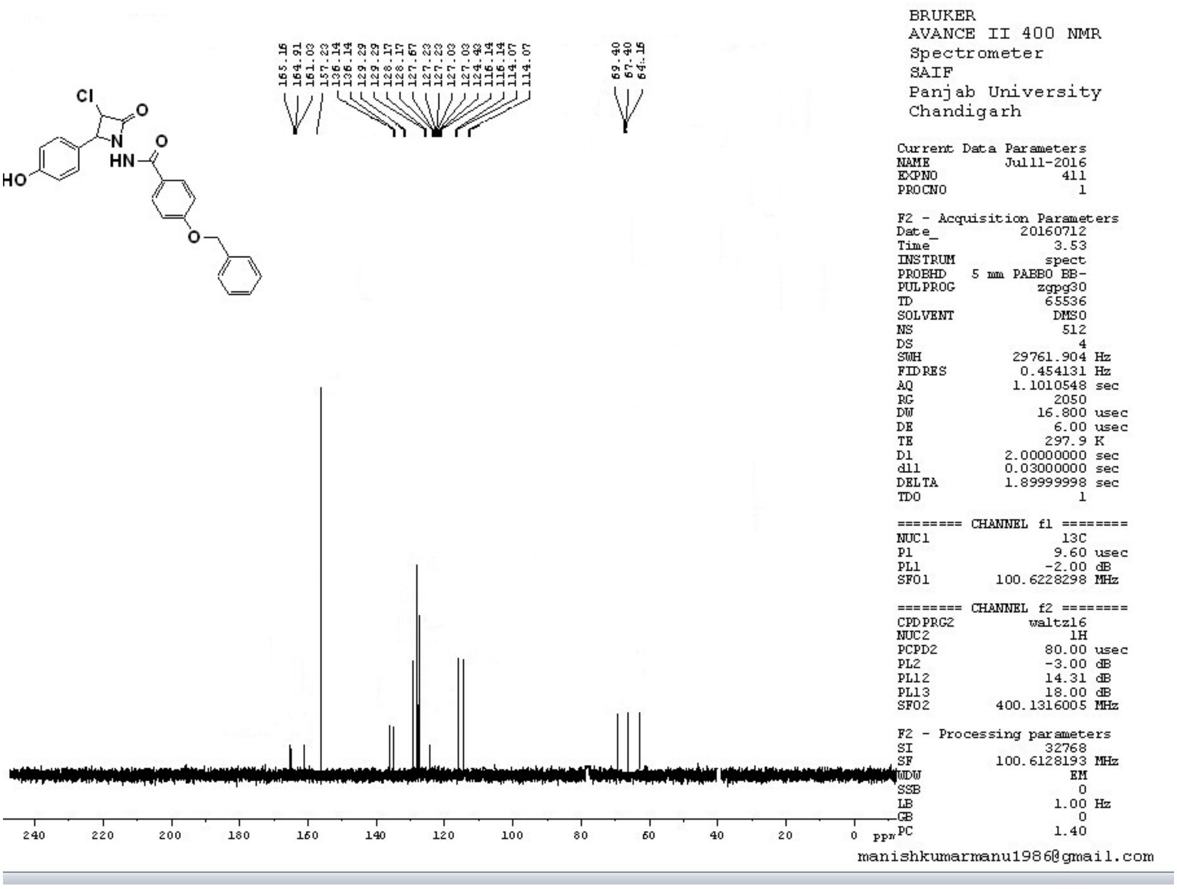

Mass Spectra 6e): ***4-(benzyloxy)-N-(3-chloro-2-(3-nitrophenyl)-4-oxoazetidin-1-yl) benzamide***

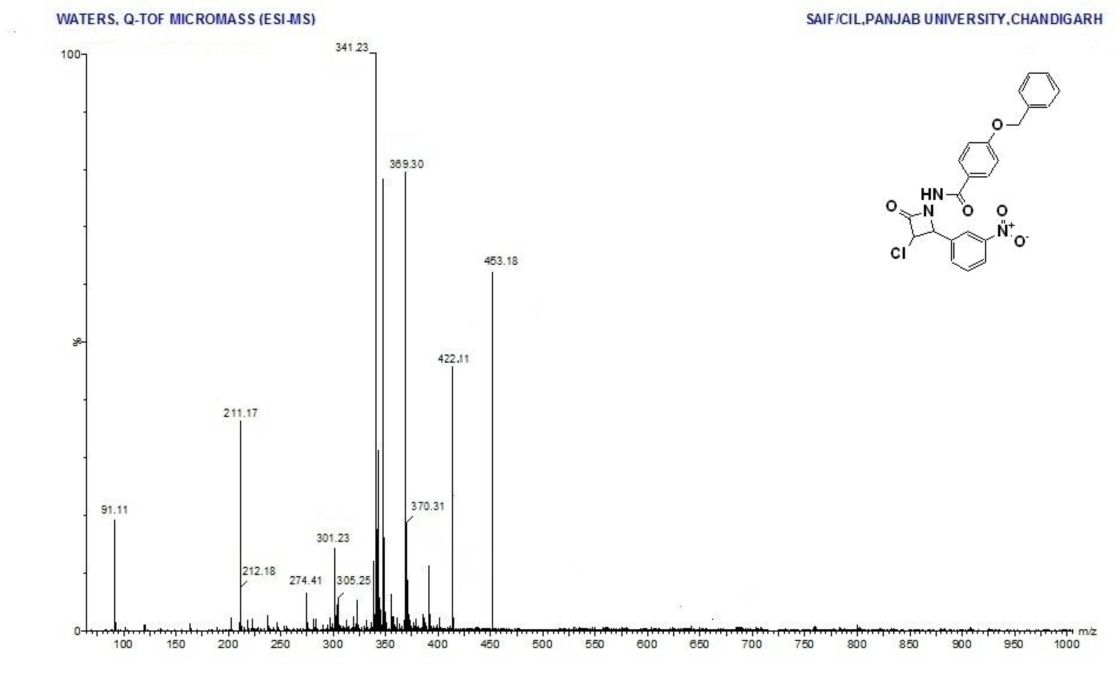

^{1}H NMR Spectra 6e): ***4-(benzyloxy)-N-(3-chloro-2-(3-nitrophenyl)-4-oxoazetidin-1-yl) benzamide***

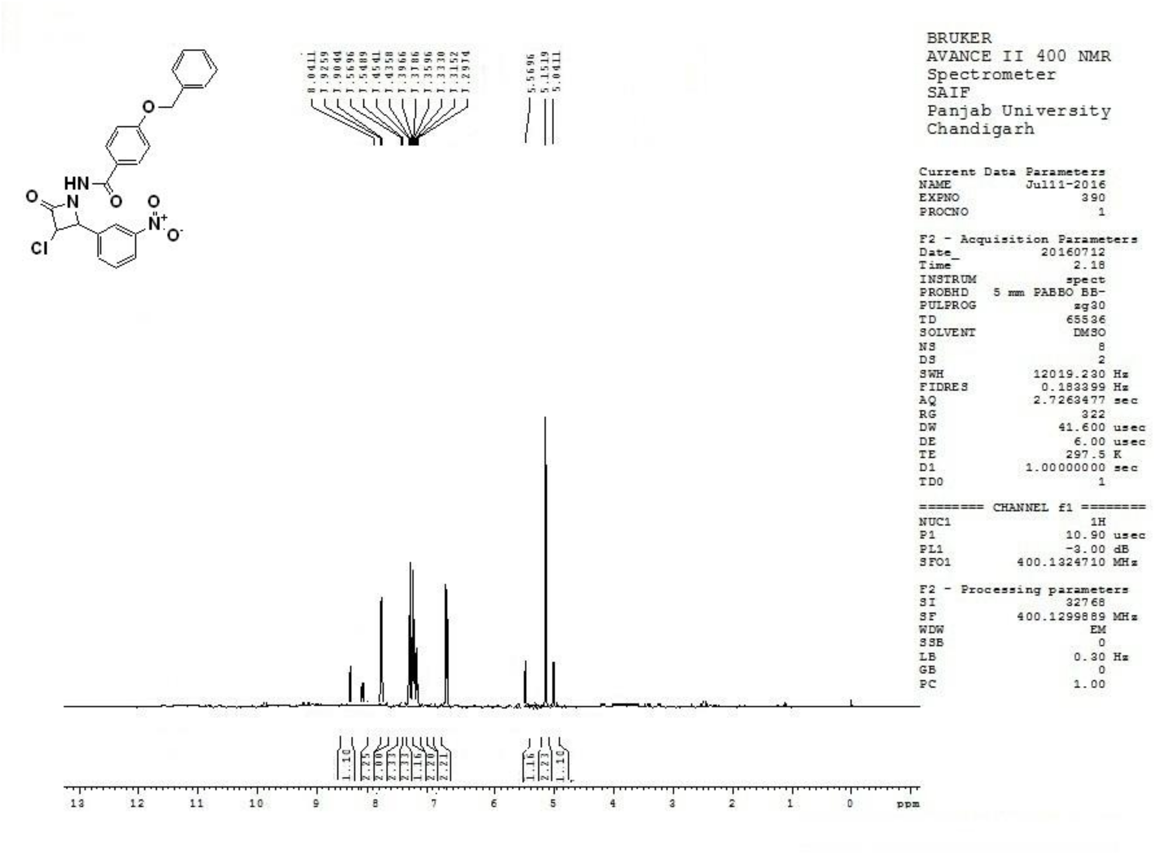

^{13}C NMR Spectra 6e): ***4-(benzyloxy)-N-(3-chloro-2-(3-nitrophenyl)-4-oxoazetidin-1-yl) benzamide***

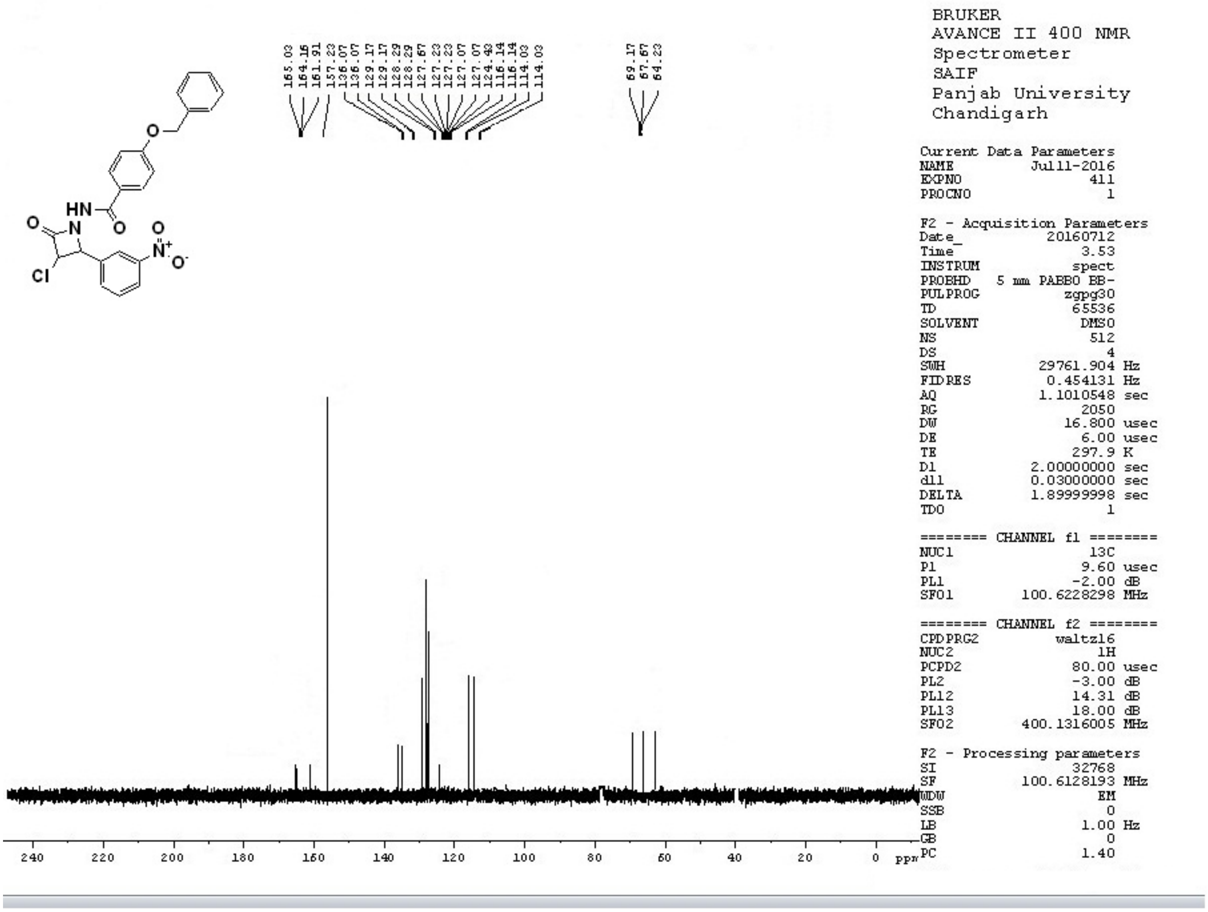

8). Elemental analysis 6j

Method name: CHNS method
Analysed: 07/01/2017 14:08
Printed: 07-01-2017 15:05
Sample ID: S
Analysis type: UnkNown
Chromatogram filename:

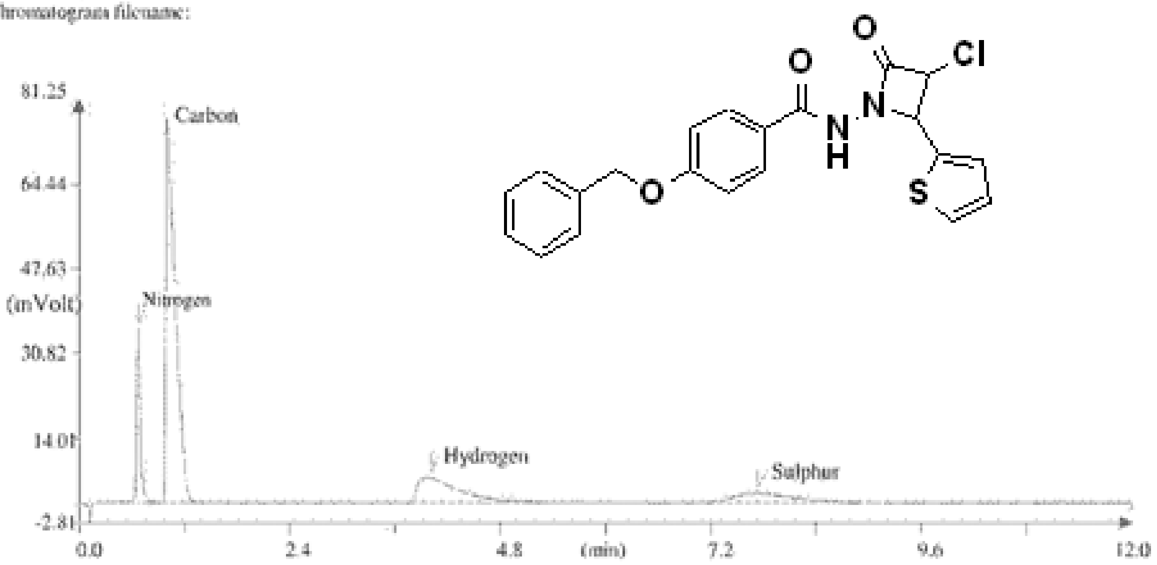

Element Name	%Element	Ret. Time	Area	BC	Area ratio	K factor
Nitrogen	7.9455	0.70	766724	RS	7.47289	0.220309E+07
Carbon	63.9767	1.07	5729644	RS	1.00000	0.475253E+07
Hydrogen	3.6536	4.00	1488423	RS	3.84947	3.141951E+08
Totals	82.5258		7985340			

S3: Structures and IUPAC name of synthesised derivatives 6 a-j

6a

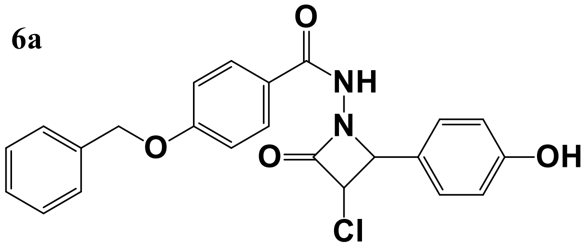

4-(benzyloxy)-*N*-(3-chloro-2-(4-hydroxyphenyl)-4-oxoazetidin-1-yl)benzamide

6b

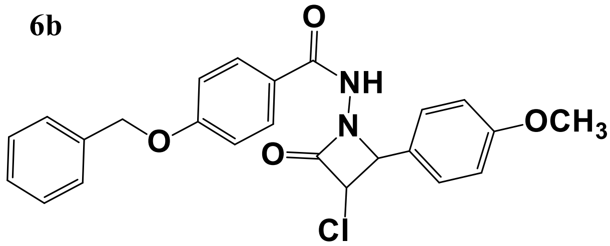

4-(benzyloxy)-*N*-(3-chloro-2-(4-methoxyphenyl)-4-oxoazetidin-1-yl)benzamide

6c

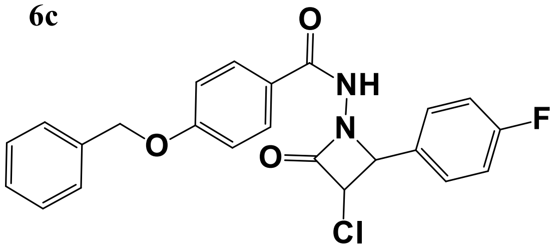

4-(benzyloxy)-*N*-(3-chloro-2-(4-fluorophenyl)-4-oxoazetidin-1-yl)benzamide

6d

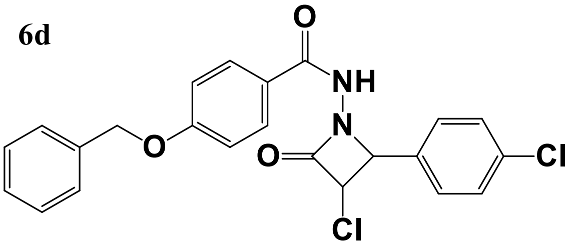

4-(benzyloxy)-*N*-(3-chloro-2-(4-chlorophenyl)-4-oxoazetidin-1-yl)benzamide

6e

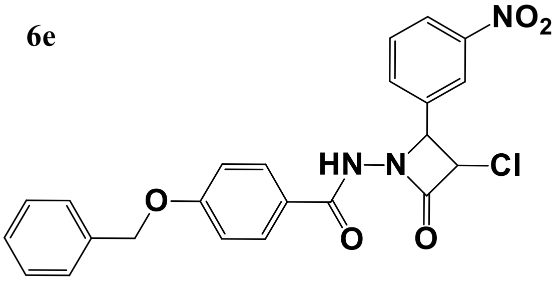

4-(benzyloxy)-*N*-(3-chloro-2-(3-nitrophenyl)-4-oxoazetidin-1-yl)benzamide

6f

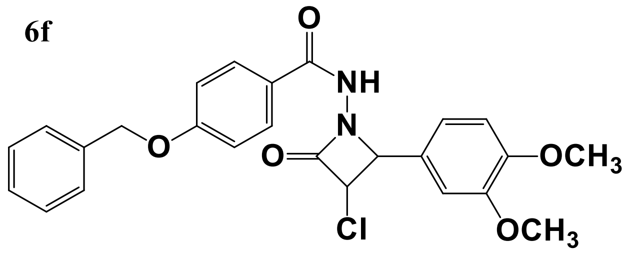

4-(benzyloxy)-*N*-(3-chloro-2-(3,4-dimethoxyphenyl)-4-oxoazetidin-1-yl)benzamide

6g

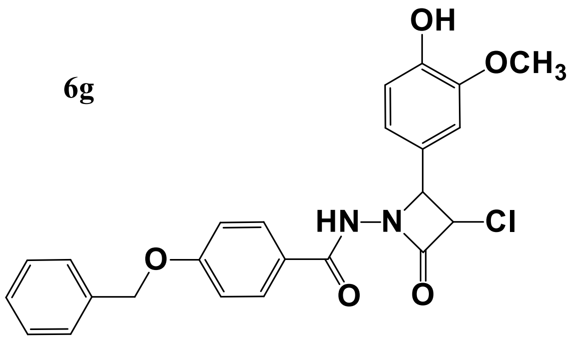

4-(benzyloxy)-*N*-(3-chloro-2-(4-hydroxy-3-methoxyphenyl)-4-oxoazetidin-1-yl)benzamide

6h

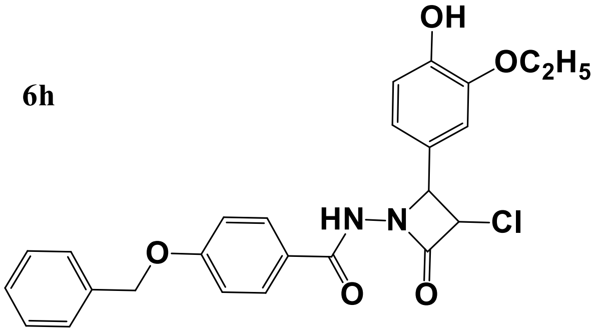

4-(benzyloxy)-*N*-(3-chloro-2-(3-ethoxy-4-hydroxyphenyl)-4-oxoazetidin-1-yl)benzamide

6i

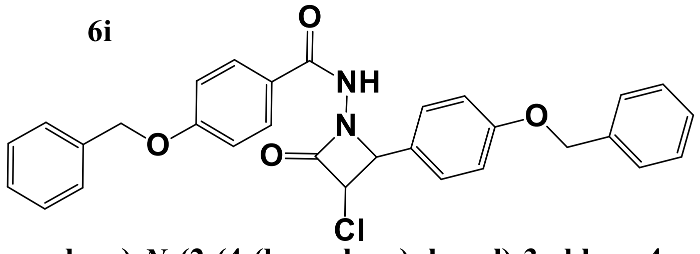

4-(benzyloxy)-*N*-(2-(4-(benzyloxy)phenyl)-3-chloro-4-oxoazetidin-1-yl)benzamide

6j

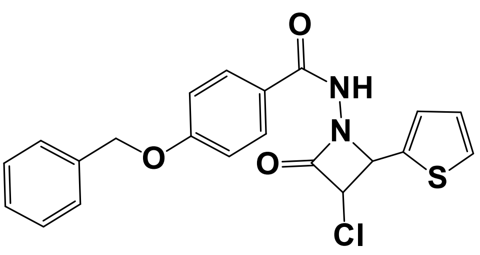

4-(benzyloxy)-*N*-(3-chloro-2-oxo-4-(thiophen-2-yl)azetidin-1-yl)benzamide
